# A standalone editing protein deacylates mischarged canavanyl-tRNA^Arg^ to prevent canavanine incorporation into proteins

**DOI:** 10.1093/nar/gkac1197

**Published:** 2023-01-11

**Authors:** Franziskus Hauth, Dietmar Funck, Jörg S Hartig

**Affiliations:** Department of Chemistry, University of Konstanz, Universitätsstraße 10, 78457 Konstanz, Germany; Konstanz Research School Chemical Biology (KoRS-CB), University of Konstanz, Universitätsstraße 10, 78457 Konstanz, Germany; Department of Chemistry, University of Konstanz, Universitätsstraße 10, 78457 Konstanz, Germany; Department of Chemistry, University of Konstanz, Universitätsstraße 10, 78457 Konstanz, Germany; Konstanz Research School Chemical Biology (KoRS-CB), University of Konstanz, Universitätsstraße 10, 78457 Konstanz, Germany

## Abstract

Error-free translation of the genetic code into proteins is vitally important for all organisms. Therefore, it is crucial that the correct amino acids are loaded onto their corresponding tRNAs. This process is highly challenging when aminoacyl-tRNA-synthetases encounter structural analogues to the native substrate like the arginine antimetabolite canavanine. To circumvent deleterious incorporation due to tRNA mischarging, editing mechanisms have evolved. However, only for half of the tRNA synthetases, editing activity is known and only few specific standalone editing proteins have been described. Understanding the diverse mechanisms resulting in error-free protein synthesis is of great importance. Here, we report the discovery of a protein that is upregulated upon canavanine stimulation in bacteria that live associated with canavanine-producing plants. We demonstrate that it acts as standalone editing protein specifically deacylating canavanylated tRNA^Arg^. We therefore propose canavanyl-tRNA^Arg^deacylase (CtdA) as systematic name. Knockout strains show severe growth defects in canavanine-containing media and incorporate high amounts of canavanine into the proteome. CtdA is frequently found under control of guanidine riboswitches, revealing a functional connection of canavanine and guanidine metabolisms. Our results are the first to show editing activity towards mischarged tRNA^Arg^ and add to the puzzle of how faithful translation is ensured in nature.

## Graphical Abstract

**Graphical Abstract:**
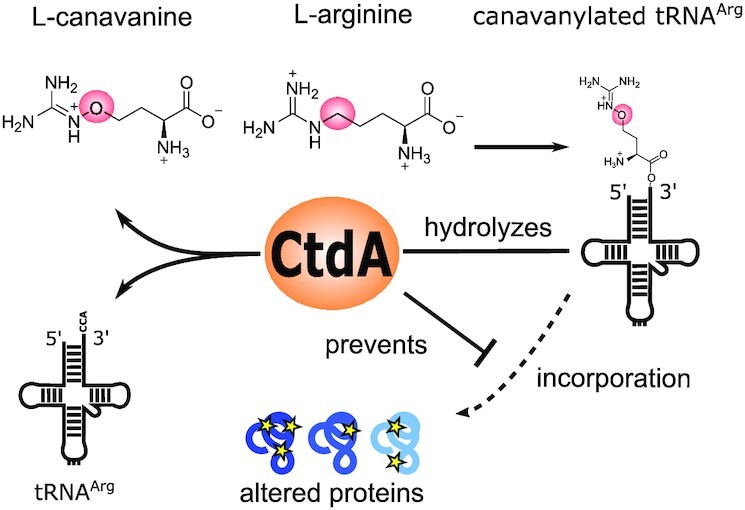
The standalone editing protein CtdA deacylates mischarged canavanyl-tRNAArg to prevent canavanine incorporation into proteins and thereby ensures translational fidelity in bacteria.

## INTRODUCTION

The translation of genetic information into functional proteins is one of the most important information transfers in all living cells. The first step in the process of protein synthesis is the activation and loading of amino acids onto their cognate tRNAs, a reaction catalysed by the family of aminoacyl-tRNA synthetases (aaRS). Two classes of aminoacyl-tRNA synthetases have evolved independently that catalyse an ATP-dependent amino acid activation followed by tRNA acylation. Each aaRS is typically specific for a single tRNA (or more, if an amino acid is encoded by several codons or several isoforms are present of a tRNA) and the matching amino acid. Ensuring the loading of the correct amino acid onto a specific tRNA is a substantial challenge, considering the number of different tRNAs and the choice between the 22 genetically encoded proteinogenic and the many different non-proteinogenic amino acids that occur in nature (1). While the selection against non-cognate tRNAs is readily accomplished due to the size of the protein-RNA contact interface (2) and specific identity elements (3), the discrimination against non-cognate amino acids is more challenging as amino acids are small molecules and only differ in their side chains. However, aaRS have evolved a double-sieve mechanism to avoid the production of mischarged tRNAs (4,5). First, aaRSs have a high specificity in the synthetic site, where the amino acids are activated by linkage to AMP and subsequently transferred to the matching tRNA. Second, several aaRS contain a proofreading (termed ‘editing’) site, that hydrolyses incorrectly loaded aminoacyl-tRNAs before they are released from the enzyme. While the first mechanism is highly selective to prevent errors, the latter exhibits low selectivity to cleave all mischarged tRNAs whereas the desired product is not cleaved (6). Discrimination and editing activities have been discovered and described in great detail in both classes of aaRS (2,6–10). Still, discrimination and editing can meet their limits, especially when the noncognate amino acid is slightly smaller than the cognate one and tRNA loading cannot be prevented on steric grounds (2). Furthermore, only half of the aaRSs have been found to display editing activity up to date (10). Impaired editing is severe and can affect cell growth or induce pathologies and apoptosis as shown for bacteria (11), yeast (12), mammalian cell culture (13) and mice (14). For several tRNAs where the aaRS does not have editing activity, autonomous standalone editing proteins have been identified in prokaryotes as well as in eukaryotes.

Standalone editing proteins catalyse *in trans* editing of mischarged tRNAs and have been identified as homologs of editing domains contained in some aaRSs. Still, the number of experimentally characterized standalone editing enzymes is very limited: ThrRS-ed is a freestanding protein (homolog of the editing domain of ThrRS) found in archaea acting on Ser-tRNA^Thr^ (15,16). AlaXps (homolog of the editing domain of AlaRS) are editing factors found in all domains of life and cleave Ser- and Gly-tRNA^Ala^ (17,18). D-aminoacyl-tRNA deacylases (DTDs) also have been shown to edit Gly-tRNA^Ala^ in multiple species (19,20), in addition to their deacylation activity of D-Tyr-tRNA^Tyr^. Finally, members of the INS superfamily (which can be divided into at least six further subgroups: INS, YbaK, ProXp-ala, ProXp-x, ProXp-ST1 and ProXp-ST2 (21)), deacylate mischarged tRNA^Pro^ (22–24). Taken together, standalone editing domain-like proteins have been described to enhance proofreading in a multitude of organisms and therefore constitute a third ‘sieve’ or mechanism of defence against accumulation of mischarged tRNAs (22).

Recently, we identified the bacterium *Pseudomonas canavaninivorans*, which can utilise canavanine as sole carbon and nitrogen source for growth (25). Canavanine, or δ-oxa-arginine, is an arginine antimetabolite used by legume plants as one of their main nitrogen storage compounds in seeds. There, canavanine is accumulated to up to 12% of the seeds’ dry weight (26). Additionally, canavanine is also exuded by the roots of young plants into the rhizosphere (27,28). In herbivores, pathogens and rhizosphere-associated bacteria, canavanine is hydrolysed by arginase, resulting in the formation of canaline that is toxic due to the formation of stable oximes with carbonyl compounds (29,30). Moreover, most ArgRSs are not able to discriminate effectively against canavanine, which leads to mischarging of tRNA^Arg^. The accumulation of canavanyl-tRNA^Arg^ then results in canavanine incorporation into nascent polypeptide chains resulting in the formation of dysfunctional proteins (31,32). In eukaryotes, the mischarging might also interfere with proteolysis as charged tRNA^Arg^ is used as a substrate for N-terminal arginylation in certain N-degrons (33,34). In accordance with its usage as antimetabolite, mechanisms to prevent auto-toxicity have been identified in canavanine-producing plants, where the discriminatory power of ArgRS towards canavanine is higher compared to non-producing plants (35). Strikingly, up to date, no editing domain in ArgRS or standalone editing proteins are described for tRNA^Arg^ (10). We gained interest in identifying such an activity because apparently *P. canavaninivorans* is able to circumvent the toxicity of canavanine. While characterizing the degradative pathway of canavanine in *P. canavaninivorans* we noted a protein annotated as B3/4-editing-domain-like protein, which was associated with the enzymes responsible for canavanine utilization (36). In addition, we noted that similar proteins are frequently controlled by two classes of guanidine riboswitches in bacteria (37–39).

## MATERIALS AND METHODS

### Oligonucleotides and chemicals

Chemicals were obtained either from Sigma-Aldrich, Roth or Acros Organics. Oligonucleotides for cloning and genotyping were purchased from Sigma-Aldrich. A list of all oligo sequences used in this study is given in the Supplementary Table S2. Radioactive [α-^32^P]- and [γ-^32^P]-ATP used for tRNA labelling was obtained from Hartmann Analytic. If not stated otherwise commercial enzymes and kits were purchased from New England Biolabs and used following the manufacturers’ instructions.

### Microbe strains

*Escherichia coli XL10* and *E. coli BL21(DE3)* cells were maintained in lysogeny broth medium at 37°C.

*P. canavaninivorans* (DSM No.: 112525) was maintained in lysogeny broth at 30°C. More detailed information on the bacterium can be found in its taxonomic and phenotypic description (25).

### Production of labelled tRNA^Arg^

tRNA was produced following the protocol by Avcilar-Kucukogze *et al.* (40). In brief, for *in vitro* transcribed tRNA two overlapping single-stranded oligonucleotides encoding the 5′ end sequence of the sense strand with an upstream T7 RNA polymerase promoter sequence and the 3′ end sequence of the antisense strand were annealed and filled using the Large Klenow Fragment of DNA polymerase. The resulting double-stranded DNA template was *in vitro* transcribed using T7 RNA polymerase followed by incubation with DNase I to digest the DNA template. The tRNA was purified by excision from 6% polyacrylamide gel electrophoresis (PAGE). Excised bands were incubated in crush and soak buffer (200 mM NaCl, 1 mM EDTA, 10 mM HEPES, pH 7.5), filtered through glass wool and tRNA was extracted by ethanol precipitation. For the over-production of tRNA^Arg^ in *P. canavaninivorans*, the tRNA^Arg^ sequence was inserted into the rhamnose-inducible expression vector pJeM1 (41) (Addgene #135088, SI Item 1)). *P. canavaninivorans* was transformed with the resulting plasmid by electroporation and transformants were selected by kanamycin resistance. A single colony was pre-cultured overnight, diluted 1:100 and grown at 30°C to an OD_600_ of 0.4. Then, rhamnose was added to induce tRNA production and the culture was grown for approximately 16 h. Cells were collected by centrifugation and tRNA was extracted as described by Avcilar-Kucukogze *et al.* (40). After extraction, the total tRNAs enriched in tRNA^Arg^ were also further purified by 6% PAGE. tRNA was 3′ α-^32^P-ATP labelled using purified *E. coli* cca tRNA nucleotidyltransferase (overexpression clone from the ASKA collection, (42)), following the protocol by Evans *et al.* (43). In summary, tRNA was first refolded by heating and slow cooling to ensure the right tRNA conformation and then treated with cca adding enzyme in a reaction where the equilibrium is shifted to exchange the terminal adenosine with α-^32^P-ATP by the addition of inorganic phosphatase and CTP. For the production of 5′ γ-^32^P labelled tRNA, unlabelled purified tRNA was first dephosphorylated using shrimp alkaline phosphatase and then labelled with γ-^32^P-ATP using T4 polynucleotide kinase following the manufacturer's instructions. In both cases labelled tRNA was purified by PAGE.

### Protein overexpression

The full length open reading frames of ArgRS (MBJ2348292.1) and B3/4 protein (MBJ2347151.1) were amplified from *P. canavaninivorans* using Phusion polymerase and cloned either via Gibson assembly or restriction digest and Quick ligation into the overexpression vector pET28a (KmR) (SI Item 2), which bears a N-terminal 6x HisTag. The resulting plasmids were amplified in *E. coli XL10* and verified by sequencing (GATC, Eurofins). The gene coding the B3/4 protein of *Clostridium perfrigens* together with restriction sites was synthesized by Twist Biosciences and cloned into pET28a using Quick ligation.

For protein overexpression, the plasmids were introduced into *E. coli BL21 (DE3)* and the respective strains were grown with suitable antibiotic overnight in lysogeny broth at 37°C, 200 rpm, diluted 1:100 and cultivated at 37°C, 200 rpm, to an OD_600_ of ∼0.5. After induction with 0.5 mM isopropyl-β-d-thiogalactopyranosid, the cultures were incubated at 18°C for ∼16 h. Cells were harvested by centrifugation and stored at –20°C. Active enzyme was purified by nickel metal affinity chromatography in gravity-flow columns. In short, frozen pellets were resuspended in lysis buffer (20 mM Tris, 20 mM imidazole, 200 mM NaCl, EDTA-free protease inhibitor (cOmplete, Roche), 0.02 mg/ml lysozyme, pH 8.0) and left on ice for 30 min. Cell lysis was completed by 6 min of ultra-sonication (1.5 s on/off cycles, 20% amplitude) on ice, after which the lysate was centrifuged to remove cell debris and insoluble material. The supernatant fraction was filtered through a 0.2 μm filter and loaded onto high performance Ni-NTA-resin. The resin was washed with wash buffer (20 mM Tris, 20 mM imidazole, 200 mM NaCl, pH 8.0) and the target proteins were eluted with elution buffer (20 mM Tris, 500 mM imidazole, 200 mM NaCl, pH 8.0). The eluted proteins were buffer exchanged into buffer without imidazole (20 mM Tris, 200 mM NaCl, pH 8.0) using PD10-desalting columns (GE Healthcare), concentrated by Amicon centrifugal filters (Merck), snap frozen in liquid nitrogen and stored at –80°C until further usage. SDS-PAGE samples were collected from all steps to monitor protein purification and integrity. A representative SDS-PAGE gel is shown in Supplementary Figure S1.

**Figure 1.**
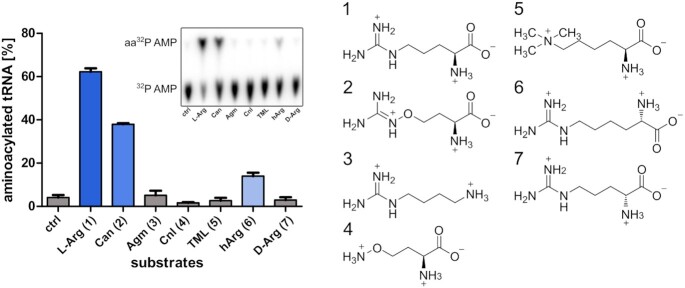
Substrate specificity of the arginine-tRNA-synthetase of *P. canavaninivorans*, error = SD of triplicates. The inlet shows a representative radio screen of the thin layer chromatography separated ^32^P AMP and aminoacylated ^32^P AMP. Pictures of all radiographs used to obtain this figure are shown in Supplementary Figure S4. 1: l-arginine, 2: l-canavanine, 3: agmatine, 4: canaline, 5: *N*-trimethyllysine, 6: homoarginine, 7: d-arginine.

To obtain amino acid-free ArgRS (following the protocol by Cvetesic *et al.* (2017)) the purified protein (20 μM) was incubated with unlabelled tRNA (20 μM) in aminoacylation reaction master mix (see section aminoacylation assay) and incubated for 15 min at 37°C. Then the reaction was incubated on ice in 2 M urea and 1 M NaCl, followed by re-purification of the enzyme via nickel metal affinity chromatography in gravity-flow columns, buffer exchange and concentration by centrifugation (all as described before).

### Aminoacylation assay

Immediately prior to the aminoacylation reaction, unlabelled tRNA was spiked with freshly prepared ^32^P labelled tRNA and then refolded. For reactions with different substrates in the presence or absence of B3/4 protein, a master mix was prepared containing ∼3 μM tRNA, 3 mM ATP, 0.0026 U/μl PPase, 0.13 μg/μl bovine serum albumin, 0.1 mM DTT, reaction buffer (50 mM HEPES, 25 mM KCl, 15 mM MgCl_2_) and 6 μM ArgRS enzyme. B3/4 protein (1 μM) was added to the master mix as indicated. Concentrations represent the final assay concentrations. The aminoacylation reactions were started by mixing 1 μl of 2 mM substrate with 5 μl of master mix followed by incubation at 37°C or room temperature for 15 min. Afterwards the reaction was quenched by the addition of 12 μl quenching buffer (1.2 M NaOAc, 0.1% [w/v] SDS, pH 4.0) and (aminoacylated) tRNA was digested using P1 nuclease for 1 h at room temperature. Then, 1 μl of digested sample was seperated by thin layer chromatography, using polyethylenimine cellulose F plates (Supelco, Merck) and 100 mM ammonium acetate in 5% (v/v) acetic acid as running buffer. After separation, the plates were dried and radiographs were recorded with a phosphorimager (GE Healthcare Life Science). Signal intensities were evaluated and quantified using ImageJ (44).

### *In-vivo* riboswitch activity assay

The guanidine class I riboswitch sequence of *Pseudomonas pelagia* CL-AP6 (DSM No. 25163, AROI01000023.1, nucleotides 57628–57864) was cloned under the control of the constitutive ON promoter lysC into the pQE vector in front of a lacZ gene. Primers and the vector map can be found in the Supplementary Information Table S2 and SI Item S3. Correct assembly of the construct was verified by sequencing and the resulting plasmid was used to transform lacZ-deficient *E. coli* strain ER2566 (NEB). Cells containing the pQE_GuaIRS_lacZ plasmid were grown overnight and then diluted to an OD_600_ of 0.05. Cells were further cultivated at 37°C until they reached an OD_600_ of ∼0.3 upon which 200 μl of culture were transferred to a 96-well plate. Candidate riboswitch ligands were added to 2.5 mM and the cultures were overlaid with 30 μl silicon oil to avoid evaporation while allowing gas exchange. Galactosidase activity assays with *o*-nitrophenyl-β-galactoside (ONPG) were conducted following the protocol by Zhang and Bremer (45) with slight modifications. The plate was further incubated at 37°C to allow expression of lacZ for 1 h after which OD_600_ was measured again and 20 μl of each sample were transferred into 80 μl permeabilization solution (100 mM Na_2_HPO_4_, 20 mM KCl, 2 mM MgSO_4_, 0.8 mg/ml CTAB, 0.4 mg/ml sodium deoxycholate, 5.4 μl/ml β-mercaptoethanol). Then, permeabilized sample (15 μl) was mixed with 90 μl of ONPG substrate solution (60 mM Na_2_HPO_4_, 40 mM NaH_2_PO_4_, 1 mg/ml ONPG, 2.7 μl/ml β-mercaptoethanol) to detect lacZ activity. ONPG solution was warmed to 30°C prior to addition. The reaction was stopped after 30 min by the addition of 105 μl 1 M Na_2_CO_3_. To evaluate colour development, the absorbance at 420 nm was measured and Miller units were calculated according to Equation (1):


(1) $$\begin{eqnarray*} && Miller\,units = 1000 \nonumber \\ && \quad *\frac{{Ab{s_{420}}}}{{\left( {\left( {O{D_{600}}\,of \, culture \,sample} \right)*\left( {volume\,\left[ {0.02\,ml} \right]} \right)*\left( {reaction\,time} \right)} \right)}} \end{eqnarray*}$$

### Homologous recombination

Gene knockouts of *P. canavaninivorans* were generated by homologous recombination as described by Huang and Wilks (46). In brief, homologous regions (500 nt up- and downstream of the gene of interest) were inserted into the pEX18Gm sacB suicide plasmid (donation from Prof. Herbert Schweizer, University of Florida, USA, SI Item 4) by Gibson assembly. The plasmid was then introduced into *P. canavaninivorans* by electroporation. Single colonies which had integrated the plasmid into the chromosome were identified by gentamicin resistance and colony PCR, followed by sacB-mediated sucrose counter-selection. Fully segregated deletion mutants were identified by the presence of a single PCR product with primers spanning the insertion site and sequencing of the respective PCR product. Additionally, the mutants were genome-sequenced (Novogene) to ensure that only the desired genes were targeted.

### Bacterial growth assays

Cells were grown in LB medium overnight and diluted to an OD_600_ of 0.005 into minimal M9 salt medium (8.5 g/l Na_2_HPO_4_·2 H_2_O, 3 g/l KH_2_PO_4_, 0.5 g/l NaCl, 1 g/l NH_4_Cl, 2 mM MgCl_2_, 100 μM CaCl_2_, trace elements (0.1 mM EDTA, 0.03 mM FeCl_3_, 6.2 μM ZnCl_2_, 0.76 μM CuCl_2_, 0.42 μM CoCl_2_, 1.62 μM H_3_BO_3_; 0.08 μM MnCl_2_), vitamins (0.1 mg/l cyanocobalamin, 0.08 mg/l 4-aminobenzoic acid, 0.02 mg/l d-(+)-biotin, 0.2 mg/l niacin, 0.1 mg/l Ca-d-(+)-pantothenic acid, 0.3 mg/l pyridoxamine-chloride, 0.2 mg/l thiamindichloride)). As carbon source, either 10 mM canavanine or 0.4% (w/v) glucose with 0, 1 or 9 mM canavanine were added from filter-sterilized stocks.

### Proteome analysis

Cells were grown overnight at 30°C in M9 salt minimal media with 0.4% (w/v) glucose as carbon source. Additionally, 3.5 mM canavanine were added to the respective samples. Cultures were harvested by centrifugation and lysed as described before, followed by centrifugation and filtration. The total protein amount was determined with the BCA Kit (Thermo Scientific) according to the manufacturer's protocol. For each proteome analysis, 50 μg of protein sample was used. The analysis was carried out by the Proteomics facility of the University of Konstanz. In short, protein samples were delivered in-gel, reduced by dithiothreitol and alkylated using chloroacetamide, followed by tryptic digest. Digested proteins were analyzed on a QExactive HF mass spectrometer (Thermo Fisher Scientific, Bremen, Germany) coupled to an Easy-nLC 1200 Nanoflow-liquid chromatography system (Thermo Fisher Scientific, Bremen, Germany). Raw data were evaluated using the software proteome discoverer 1.4 (Thermo Fisher scientific) and peptides were identified by comparison to the predicted proteome of *P. canavaninivorans (**25**)*.

## RESULTS

### ArgRS does not discriminate efficiently against canavanine

We used the tRNAscan-SE server (47) to search for potential tRNA^Arg^ in the genome of *P. canavaninivorans* and identified three candidate tRNAs. We then *in vitro*-transcribed and ^32^P-labelled the tRNAs and performed aminoacylation reactions using the ArgRS of *P. canavaninivorans*, which was overexpressed and purified from *E. coli*. We observed successful arginylation for two of the tRNAs and to a lesser extend also canavanylation (Supplementary Figure S2). To further test the specificity of the ArgRS we tested several structurally similar compounds as potential substrates. As arginine was tightly binding to ArgRS during protein purification, we used the protocol described by Cvetesic *et al.* (9) to obtain amino acid-free ArgRS. We observed aminoacylation with arginine and canavanine and to a lesser degree with homoarginine, but no reaction for the negative control agmatine and the substrate analogs canaline, trimethyllysine and d-arginine (Figure 1). Next, we determined the Michaelis-Menten-kinetic parameters of ArgRS with the substrates arginine and canavanine (Supplementary Figure S3) to clarify whether the native arginine tRNA synthetase in *P. canavaninivorans* possesses sufficient discriminatory power to prevent potentially harmful canavanyl-charging activity of tRNA^Arg^. Remarkably, the observed *K*_M_s of the two substrates were very similar (5.9 ± 1.2 and 23.6 ± 9 μM), while the observed *v*_max_ differed by a factor of 3. Hence, the discriminatory factor *D* ((*k*_cat_/*K*_M_ (arginine))/(*K*_cat/_*K*_M_ (canavanine))) was calculated to be 12.9. A discriminatory factor of 3000 is assumed to be the lower threshold where mis-incorporation of the non-cognate amino acid does not impact cellular fitness (48–50). Consequently, our finding strongly suggests the need for editing activity towards canavanyl-tRNA^Arg^ in bacteria that are exposed to canavanine. Moreover, the reported half life of canavanyl-tRNA^Arg^ is ∼5 min compared to ∼46 min for arginine-tRNA^Arg^ (35), indicating that there might be even less discriminatory potential due to the faster decay of canavanyl-tRNA^Arg^ resulting in a lower observed *v*_max_.

### Comparative proteomics reveals the upregulation of a standalone B3/4 editing domain-like protein upon canavanine stimulation

**Figure 2.**
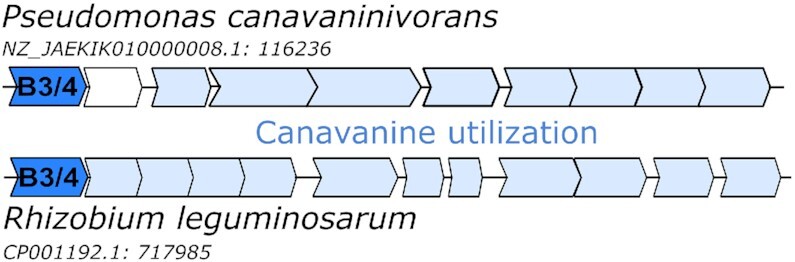
Position of the B3/4 protein in the genome of *P. canavaninivorans* and *R. leguminosarum* relative to the canavanine degradation operon.

To identify mechanisms that could avoid canavanine incorporation into proteins, we carried out comparative proteomics of *P. canavaninivorans* cultures grown on canavanine or glycerol as sole carbon source. Apart from the canavanine utilization pathway described by us recently (36), a protein annotated as B3/4 editing domain-like protein (NCBI MBJ2347151.1, from here on called B3/4 protein) was only detectable in the canavanine-grown culture and not in the culture grown on glycerol (see Supplementary Table S1). The annotation refers to the similarity to the B3 and B4 domains of the phenylalanine tRNA synthetase subunit β, which were identified to form the editing site of the enzyme (8). Interestingly, we found the B3/4 protein located right in front of the canavanine utilization operon. We previously studied canavanine utilization also in a second bacterium, *Rhizobium leguminosarum*. There, we also found a homolog of the B3/4 protein (55% identity, 68% similarity) co-localized with canavanine degradation genes (Figure 2). Hence, we speculated that this protein is responsible for correcting the mis-charging of tRNA^Arg^ with canavanine. *P. canavaninivorans* as well as *R. leguminosarum* are both natural habitants of the canavanine-rich legume rhizosphere and therefore most likely adapted to circumvent its toxicity.

### The B3/4 protein edits canavanylated tRNA^Arg^*in vitro*

**Figure 3.**
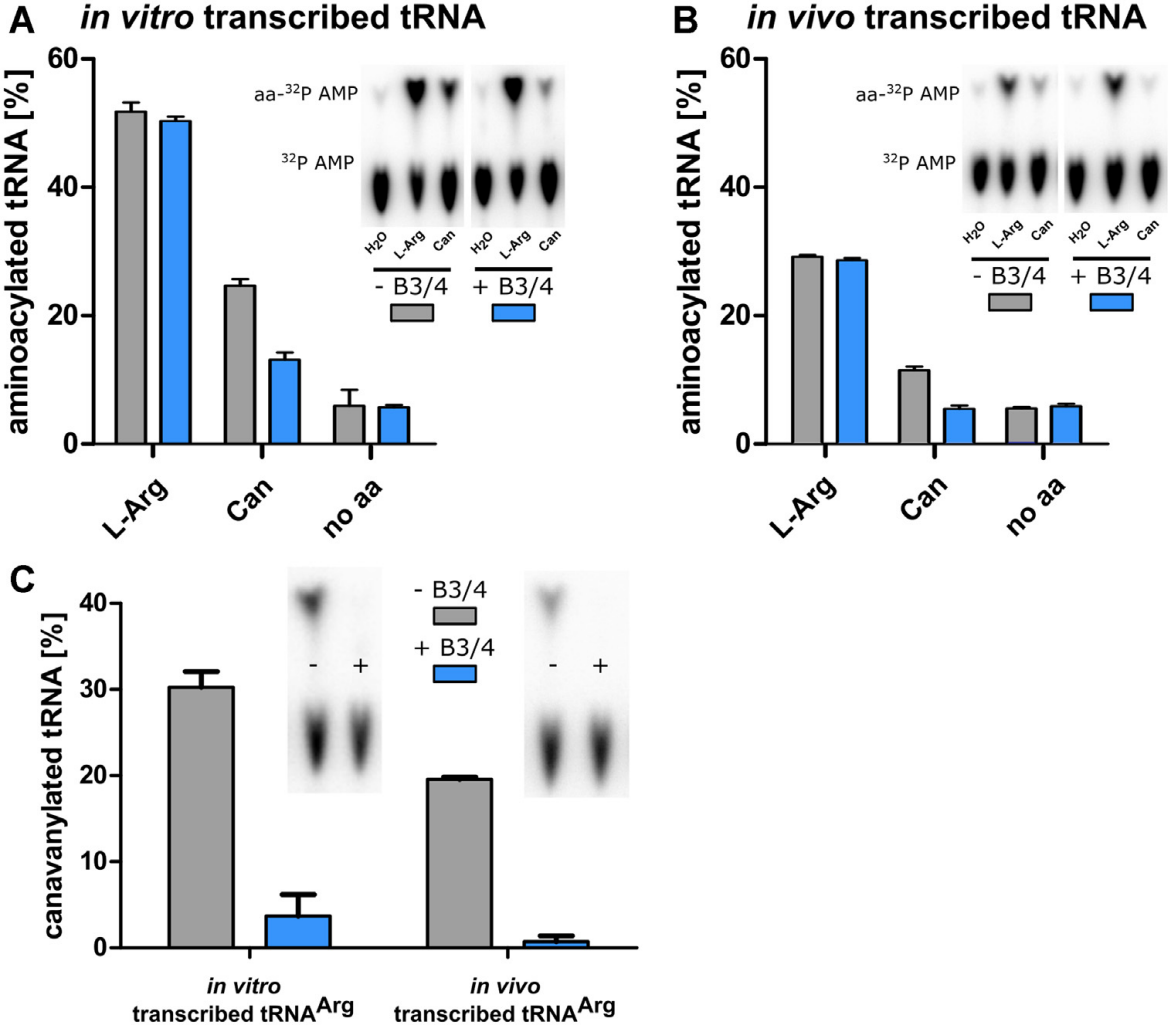
Deacylase activity of the B3/4 protein. (**A**) Level of *in vitro*-produced aminoacylated tRNA^Arg^ in the presence (blue) or absence (grey) of B3/4 protein together with either arginine, canavanine or no amino acid (aa), error = SD of triplicates, the B3/4 protein was added to the ongoing aminoacylation reaction. The inlet shows a representative radiograph of the thin layer chromatography-separated ^32^P-AMP and aminoacylated ^32^P-AMP, all radio screen images can be found in the SI. (**B**) Same as (A) but using *in vivo*-produced tRNA. (**C**) Level of canavanylated tRNA after 1 min incubation with (blue) or without (grey) B3/4 protein, the protein was added after the aminoacylation reaction was quenched and the canavanylated tRNA was purified, error = SD of triplicates. Pictures of all radiographs used to obtain the figure are shown in the Supplementary Figures S4–S6.

Upon addition of the B3/4 protein to the aminoacylation reaction with ArgRS, less canavanylated tRNA^Arg^ was observed, while there was no effect on obtained levels of arginyl-tRNA^Arg^ (Figure 3A) indicating that the B3/4 protein can edit canavanyl-tRNA^Arg^ or stimulate editing activity in ArgRS. As the natural modifications of tRNA^Arg^ could play an important role in the recognition process by ArgRS or B3/4 protein and thereby influence aminoacylation and editing, we carried out the aminoacylation reaction with tRNA^Arg^ overexpressed in *P. canavaninivorans*. The *in vivo-*produced tRNA comprises a pool of all expressed tRNAs with the desired tRNA^Arg^ enriched by overexpression, which explains the changes in the overall charging levels. Also, editing of canavanyl-tRNA^Arg^ by the B3/4 protein appeared to be more effective compared to the reaction with *in vitro*-transcribed tRNA^Arg^ (Figure 3B), but the effect is most likely due to a decreased activity of ArgRS due to the additional tRNA modifications. If the rate of aminoacyl-tRNA synthesis is decreased, post-transfer editing becomes more effective (51). The observed level of canavanylated tRNA upon addition of B3/4 protein was as low as the background without added amino acid, whereas the arginylation was again unaffected. Also, no editing activity was apparent for homoarginylated tRNA^Arg^ (Supplementary Figures S2 and S5), indicating that the B3/4 protein activity might be specific for canavanyl-tRNA^Arg^.

To clarify if the editing protein needs ArgRS or if it has a standalone catalytic activity we incubated purified canavanyl-tRNA^Arg^ with the B3/4 protein. To compensate for the instability of canavanyl-tRNA^Arg^ (35), we incubated the samples for only 1 min and added B3/4 protein in high excess (50 μM protein to 3 μM canavanylated tRNA^Arg^) to achieve rapid deacylation. After incubation with B3/4 protein, almost complete deacylation of canavanylated tRNA^Arg^ was observed for *in vitro* as well as *in vivo*-produced tRNA^Arg^ (Figure 3C), indicating that ArgRS is not needed for editing activity.

### A guanidine-I riboswitch-associated B3/4 protein also prevents canavanylation of tRNA^Arg^

**Figure 4.**
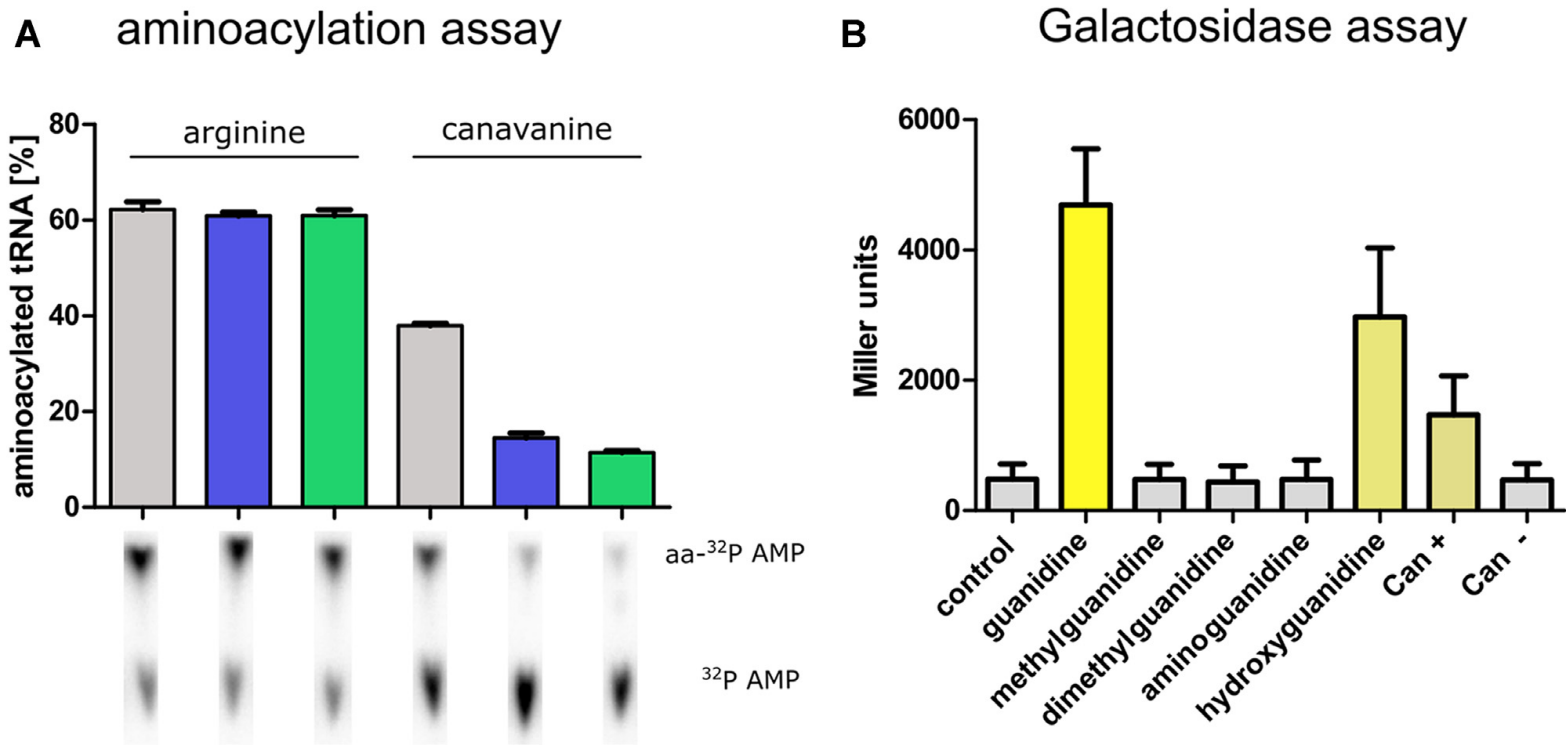
Activity of a distantly related B3/4 protein and riboswitch-dependent gene regulation. (**A**) Level of *in vitro* produced aminoacylated tRNA^Arg^ in the presence (blue: *P. canavaninivorans*, green: *C. perfrigens*) or absence (grey) of B3/4 protein, error = SD of triplicates. Pictures of all radiographs used to obtain this figure are shown in Supplementary Figure S4. (**B**) ONPG assay of a guanidine class I riboswitch (*P. pelagia*) exerting control over lacZ. Cells were grown in the presence of 2.5 mM of the indicated compound. Can: canavanine, + indicates that canavanine was incubated with the enzyme CanγL resulting in the formation of hydroxyguanidine and homoserine.

Next, we wondered if the B3/4 protein was a specific feature of legume-associated bacteria like *P. canavaninivorans* or *R. leguminosarum* or if the protein is further found in other bacteria. For this reason, we tested whether a distant homolog of the protein (35% sequence identity) also hydrolyses canavanyl-tRNA^Arg^. The B3/4 protein found in *Clostridium perfringens* (WP_004456252.1) indeed prevented the accumulation of canavanylated tRNA^Arg^ in the aminoacylation assay (Figure 4A). Interestingly, the *C. perfringens* B3/4 protein and many other B3/4-annotated proteins are found to be controlled by guanidine class I and class IV riboswitches (37–39) and the corresponding bacteria are mostly found in microoxic or anaerobic habitats like the gut of higher animals where canavanine exposure could occur via the ingestion of canavanine-producing legumes. The observation that B3/4 proteins involved in canavanine metabolism are found under control of guanidine riboswitches is unexpected but could hint at the possibility that the presence of guanidine acts as an indicator for canavanine exposure. Since canavanine is structurally very similar to arginine, it might be more easy to sense a specific degradation product of canavanine instead of the molecule itself. Guanidine detection would then indicate the presence of canavanine, a potentially toxic antimetabolite that warrants additional measures such as an increased proofreading of tRNA^Arg^.

Two early studies reported the degradation of canavanine to homoserine and guanidine (52) as well as to homoserine and hydroxyguanidine (53). We recently identified and confirmed the latter degradation pathway and characterized the responsible enzymes with canavanine-γ-lyase as key activity in the newly identified bacterium *P. canavaninivorans* (36). In light of the above-mentioned hypothesis that guanidine could act as an indicator compound for the presence of canavanine, we wondered whether hydroxyguanidine, the so-far confirmed degradation product of canavanine, would also trigger a guanidine riboswitch. Especially guanidine class I riboswitches have been reported to be highly specific with regard to guanidine recognition, even rejecting substrates that are structurally very similar, such as aminoguanidine and methylguanidine (37). In order to characterize the response to hydroxyguanidine, a Gd-I riboswitch from *Pseudomonas pelagia* was inserted into the 5′-UTR of a lacZ gene and its expression in a lacZ-deficient *E. coli* strain in the presence of potential riboswitch ligands was measured using ONPG as substrate. Interestingly, in addition to the expected guanidine also hydroxyguanidine triggered a riboswitch response (Figure 4B) whereas other close analogs did not show induction of gene expression. In addition to using commercial hydroxyguanidine, we also produced it from canavanine by pre-incubation with canavanine-γ-lyase (CanγL, Can+/Can– in Figure 4B). Enzymatically produced hydroxyguanidine also triggered the riboswitch. The finding supports our hypothesis that in some cases guanidine or hydroxyguanidine could act as indicators for the presence of guanidine-producing compounds such as canavanine.

### The B3/4 protein mitigates canavanine toxicity *in vivo*

**Figure 5.**
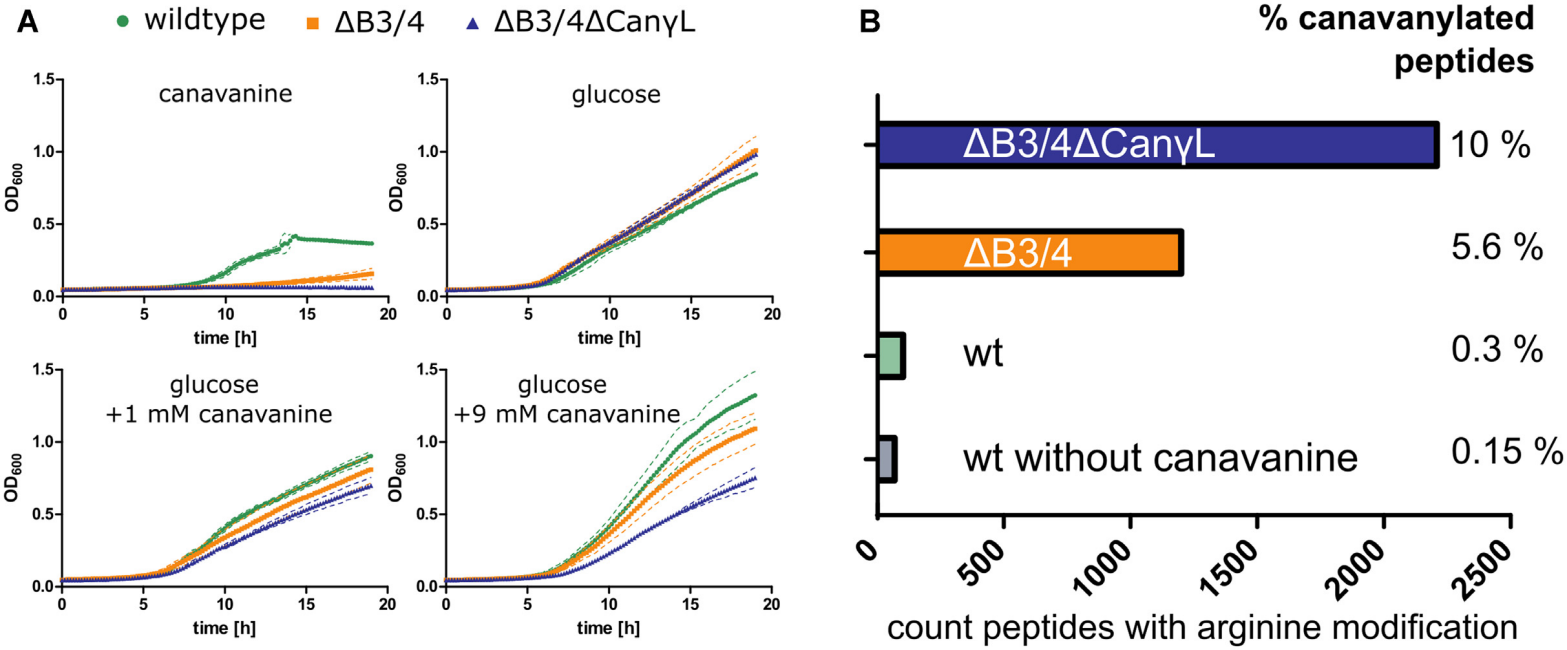
Relevance of canavanyl-tRNA^Arg^-editing *in vivo*. (**A**) Growth curves of *P. canavaninivorans* wt, ΔB3/4 and ΔB3/4 ΔCanγL strains grown in minimal M9 medium with either canavanine, glucose or glucose + canavanine as carbon source, dashed line = SD of triplicates. (**B**) Peptides counted with arginine modifications and missed cleavages in a whole proteome analysis. Indicated bacterial strains were grown in minimal M9 medium with glucose and 3.5 mM canavanine, ‘% canavanylated peptides’ shows the number of peptides found to be mass-shifted in combination with a missed tryptic cleavage compared to the overall found peptides in the sample. For more details, see Supplementary Table S3.

To elucidate a potential impact of the B3/4 protein′s activity on bacterial viability and growth, we generated a *P. canavaninivorans* ΔB3/4 deletion strain by homologous recombination. Additionally, we created a strain (ΔB3/4 ΔCanγL) where we additionally deleted the recently characterized canavanine-γ-lyase (36) as we reasoned that this would render *P. canavaninivorans* unable to detoxify canavanine by degradation, thereby promoting its potentially deleterious effect. In M9 minimal medium with canavanine as sole carbon source, we observed that the wildtype (wt) strain was able to grow, while expectedly the double deletion strain ΔB3/4 ΔCanγL was unable to grow on canavanine alone (Figure 5A). In accordance with the proposed editing activity, the ΔB3/4 protein single deletion strain was strongly impaired in growth in comparison to the wildtype strain. To exclude a general growth deficit due to the gene deletions, we cultivated the three strains in minimal medium with glucose as carbon source supplemented with different levels of canavanine. In the absence of canavanine we did not observe notable differences in growth, whereas upon addition of 1 mM canavanine, growth rates differed between the strains, and this effect became more pronounced upon addition of 9 mM canavanine. In the presence of canavanine, wildtype *P. canavaninivorans* grew better than the two mutant strains and among these the double deletion strain grew worst.

### B3/4 protein prevents incorporation of canavanine into the proteome

**Figure 6.**
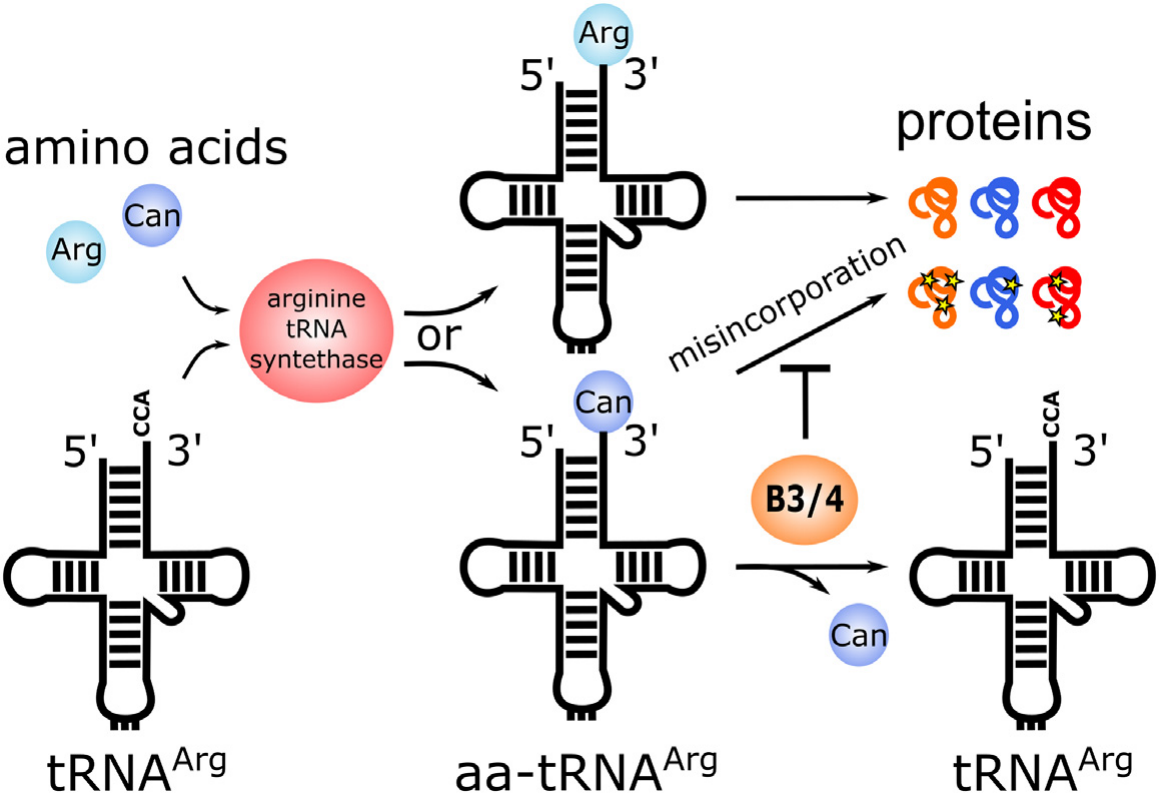
Scheme of the proposed activity of CtdA. Arg = arginine, Can = canavanine. Stars represent canavanine incorporation into proteins, which is presumably toxic to the cell.

To evaluate if the growth impairment was due to the incorporation of canavanine into the proteome and whether the activity of the B3/4 protein is impacting the proteome composition in presence of canavanine, we grew the wildtype and the two mutant strains in canavanine-containing M9 minimal medium and analysed their proteomes by mass spectrometry to investigate whether canavanine was incorporated into proteins instead of arginine. The incorporation of canavanine was detected during data evaluation as an arginine modification with a mass shift of +2. Additionally, incorporation of canavanine instead of arginine almost completely blocks the tryptic digest during sample preparation (see Supplementary Table S3 and Crine and Lemieux (54)). The combination of mass shift and missed cleavage hence provides a reliable way to identify misincorporation of canavanine. We observed that only 0.15% of the detected peptides matched both criteria (mass shift + missed cleavage) in the wt culture without canavanine, whereas in a wt culture with 3.5 mM canavanine the percentage already doubled to 0.3%. In the single deletion strain ΔB3/4 we found ∼5.6% of all detected peptides canavanylated, which corresponds to a 19-fold change compared to wt, while in the double deletion strain ΔB3/4 ΔCanγL the effect was even more pronounced (10% canavanylated peptides, 33-fold compared to wt) (Figure 5B). The proteome data provides further evidence that the B3/4 protein prevents canavanine incorporation into proteins via deacylation of canavanylated tRNAs. A scheme of the proposed function of B3/4 is presented in Figure 6.

## DISCUSSION

In this work, we describe a standalone protein annotated as B3/4 editing domain-like protein that specifically deacylates canavanyl-tRNA^Arg^. The enzyme was found upregulated when the bacterium *P. canavaninivorans* grew on canavanine as sole carbon source (36). By aminoacylation experiments, we showed that ArgRS of *P. canavaninivorans* does not discriminate sufficiently between arginine and canavanine and that the B3/4 protein is editing canavanyl-tRNA^Arg^ as a standalone protein. We therefore propose to rename this specific class of B3/4 proteins to canavanyl-tRNA^Arg^deacylase (CtdA). Growth experiments with *P. canavaninivorans* gene deletion strains confirm the physiological importance of B3/4 editing activity and proteome data strongly suggest that impaired growth of the deletion strains is due to massive canavanine misincorporation into the proteome. A homolog of CtdA is also found in other rhizosphere inhabitants like *R. leguminosarum* where it is similarly located directly upstream of an operon coding for a canavanine degradation pathway. We conclude that the enzymatic activity of CtdA appears to be part of the bacterial response to encountering canavanine.

Sequence comparison and direct enzymatic assays of a distantly related protein under control of a guanidine class-I riboswitch identified an additional group of tRNA^Arg^ editing factors. The occurrence of guanidine riboswitch-mediated control of CtdA expression hints at an intricate linkage between canavanine and guanidine metabolisms, potentially by sensing guanidine as an indicator compound signalling the presence of guanidine-generating metabolites such as canavanine that can be erroneously loaded onto tRNA^Arg^. The gut inhabitant *E. faecalis* was shown to reduce canavanine to homoserine and guanidine (52), however no molecular characterization of this activity has been reported up to date. The finding that also hydroxyguanidine triggered the guanidine-I riboswitch makes sense in light of our recent report of a canavanine degradation pathway that starts with the hydrolysis of canavanine to homoserine and hydroxyguanidine (36). The hypothesis that guanidine and hydroxyguanidine are detected by riboswitches as indicator compounds for the presence of toxic antimetabolites such as canavanine that are not easily detectable due to structural similarity to arginine adds another physiological function of guanidine sensing. Originally, guanidine sensing by riboswitches has been postulated to serve the purpose of inducing mechanisms to detoxify guanidine by export or degradation (37). Additionally, we found that riboswitch-induced guanidine degradation activities serve the purpose of assimilating the nitrogen-rich compound (55,56).

The finding of a standalone enzyme able to discriminate arginyl-tRNA^Arg^ and its close structural analog canavanyl-tRNA^Arg^ is in itself remarkable. As mentioned before, the acylated tRNAs are very similar with regard to their size and shape. However, the electron-withdrawing effect of the oxa-group in canavanine changes the p*K*_a_ of the guanidinium group drastically from 13.8 to approximately 7 (57,58). A potential basis for discrimination could hence be the different binding of charged and uncharged guanidine residues where only the substrate complex with the uncharged sidechain results in catalytic activity. Moreover, the oxaguanidine group of canavanine could potentially take part in the catalytic mechanism in a co-factor-like manner. Due to the lowered p*K*_a_, the canavanine sidechain could engage in general acid-base catalysis to support cleavage of the ester bond between canavanine and the tRNA. These speculations offer a promising starting point for a more thorough investigation of the catalytic mechanism of this novel subclass of tRNA deacylases with a potentially distinct reaction mechanism.

## DATA AVAILABILITY

All relevant data are available in the manuscript and the supplementary materials.

## Supplementary Material

gkac1197_Supplemental_Files
