# Supplementary material for: A standalone editing protein deacylates mischarged canavanyl-tRNAArg to prevent canavanine incorporation into proteins
Source: Nucleic Acids Res. 2023 Jan 11;51(5):2001–10. doi: 10.1093/nar/gkac1197 (PMC10018355; doi:10.1093/nar/gkac1197)
Supplement: gkac1197_Supplemental_Files [file gkac1197_supplemental_files.zip › 221115_revised SI.pdf]

Supplementary information for

A standalone editing protein deacylates mischarged canavanyl-tRNA^{Arg} to prevent canavanine incorporation into proteins

Franziskus Hauth[1,2], Dietmar Funck[1] and Jörg S. Hartig[1,2*]

1: Department of Chemistry, University of Konstanz, Universitätsstraße 10, 78457 Konstanz, Germany

2: Konstanz Research School Chemical Biology (KoRS-CB), University of Konstanz, Universitätsstraße 10, 78457 Konstanz, Germany

*To whom correspondence should be addressed. Tel: +49 7531 88 4575; Email: joerg.hartig@uni-konstanz.de

Content:

SI Figure 1

SI Figure 2

SI Figure 3

SI Figure 4

SI Figure 5

SI Figure 6

Additional supporting material as separate files:

SI Tables: “SI Tables 1 to 3”

SI Item 1: “SI Item 1_pJeM1”

SI Item 2. “SI Item 2_pET28a”

SI Item 3: “SI Item 3_pQE_lysC_promoter”

SI Item 4: “SI Item 4_pEX18GM”

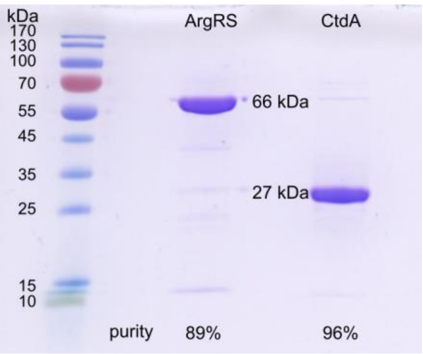

SI Figure 1: Representative SDS-PAGE of proteins ArgRS and CtdA after purification stained with Comassie Blue. Purity was determined using ImageJ.

$tRNA^{Arg}$ 1: 5’ – GTCTCAGTAGCTCAATTGGATAGAGCATCCCCCT**CCT**AAGGGGAAGGTTGGCAGTTCGAACCTGCCCTGGGACACCA – 3'

$tRNA^{Arg}$ 2: 5’ – GCACCAGTAGCTCAGCTGGATAGAGTACTGCCCT**CCG**AAGGCAGGGGTCGTGGGTTCGAATCCCGCCTGGTGCACCA – 3'

$tRNA^{Arg}$ 3: 5’ – GCGCCCGTAGCTCAGCTGGATAGAGCATCCGCCT**TCT**AAGCGGATGGTCGCAGGTTCGAGTCCTGCCGGGTGCGCCA – 3'

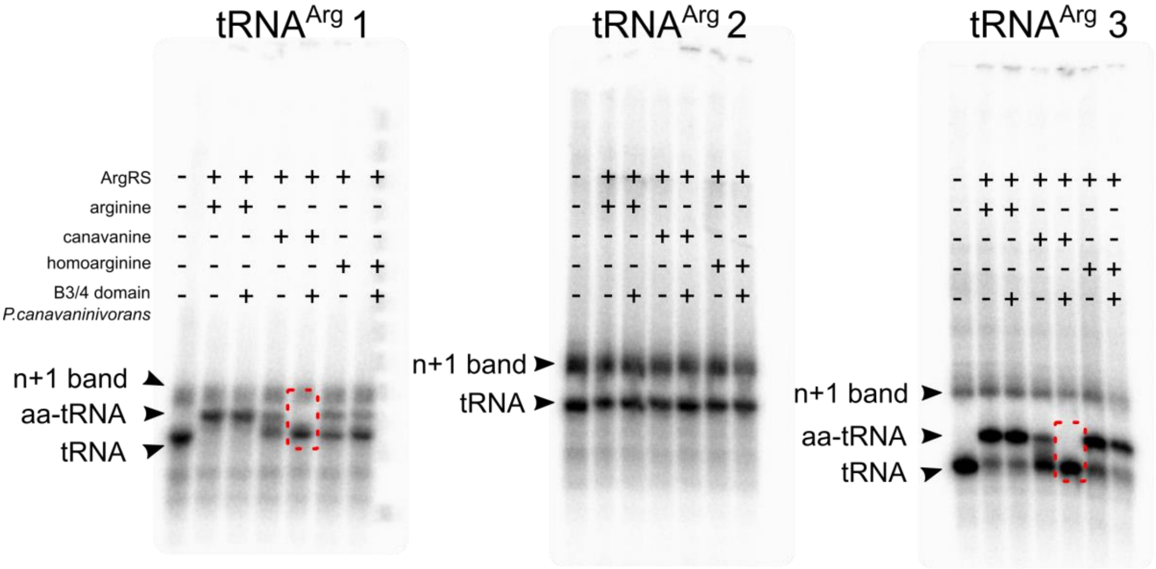

SI Figure 2: Radio screen of an acid PAGE separating the 5’ end γ-^{32}P labelled (aminoacylated) tRNAs. The sequences of the predicted tRNAs are shown above. The n+1 band is a common artefact of *in vitro* produced RNAs, but can be reduced by using modified primers. Aminoacylation reactions were performed in the presence or absence of the respective substrate and B3/4 domain like protein for 2h at 37°C. Only canavanylated but neither arginylated or homoarginylated tRNA was hydrolysed by the B3/4 domain like protein, see red box.

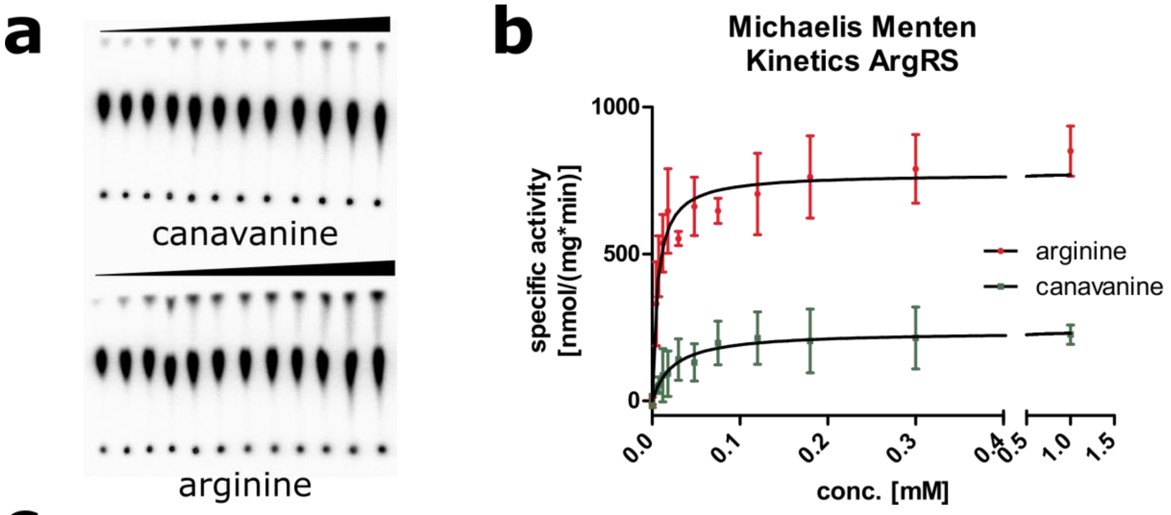

c

	arginine	canavanine		arginine	canavanine
v_{max} (obs) [nmol/(min*mg)]	773.8 ± 28.3	235.8 ± 23.5	k_{cat} (obs) [s^{-1}]	0.86 ± 0.03	0.26 ± 0.03
K_M (obs) [µM]	5.9 ± 1.2	23.6 ± 9.0	k_{cat} (obs)/K_M (obs) [s^{-1}/nM^{-1}]	143.9	11.1
			D (Arg/Can)	12.9	

SI Figure 3: a: Representative radio screen of TLC separated 3' ^{32}P labelled (aminoacylated) AMP. Either arginine or canavanine were added in rising concentrations (0, 4.8, 7.5, 12, 18, 30, 38, 75, 120, 180, 300, 1000 µM) to the acylation reaction. **b:** Reaction velocity plotted over substrate concentration with fit, assuming Michaelis-Menten Kinetics, error = S, n = 3. **c:** Observed Michelis-Menten parameters used for the calculation of the discrimination factor D (k_{cat}/K_M (Arg) divided by k_{cat}/K_M (Can)).

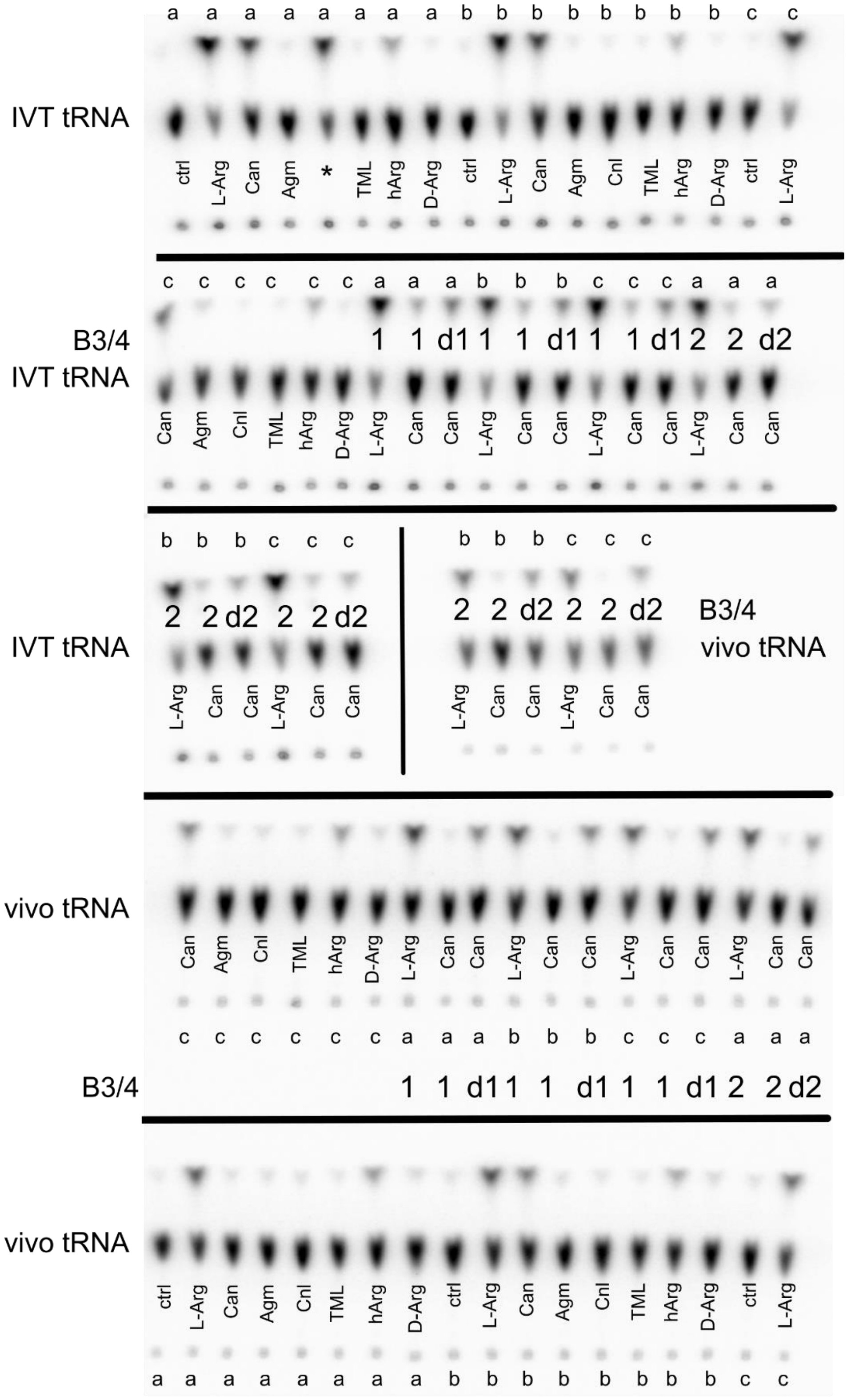

SI Figure 4: Radio screens of TLC separated 3’ ^{32}P (aminoacylated) AMP of triplicate experiments (a, b, c). Either *in vitro* or *in vivo* produced tRNA was used in the aminoacylation experiment, which was conducted in the presence of B3/4 domain like protein as indicated. B3/4 1 represents the domain from *P. canavaninivorans*, 2 the domain from *C. perfrigens*, d1/d2 indicates that the domain was heat incubated prior to the aminoacylation reaction, the substrate used in the reaction is shown above the TLC loading spot: ctrl: H_2O, Arg: L-Arginine, Can: canavanine, Agm: negative control agmatine, Cnl: canaline, TML: N-trimethyllysine, hArg: homoarginine, D-Arg: D-arginine, * pipetting error during spotting on the TLC plate.

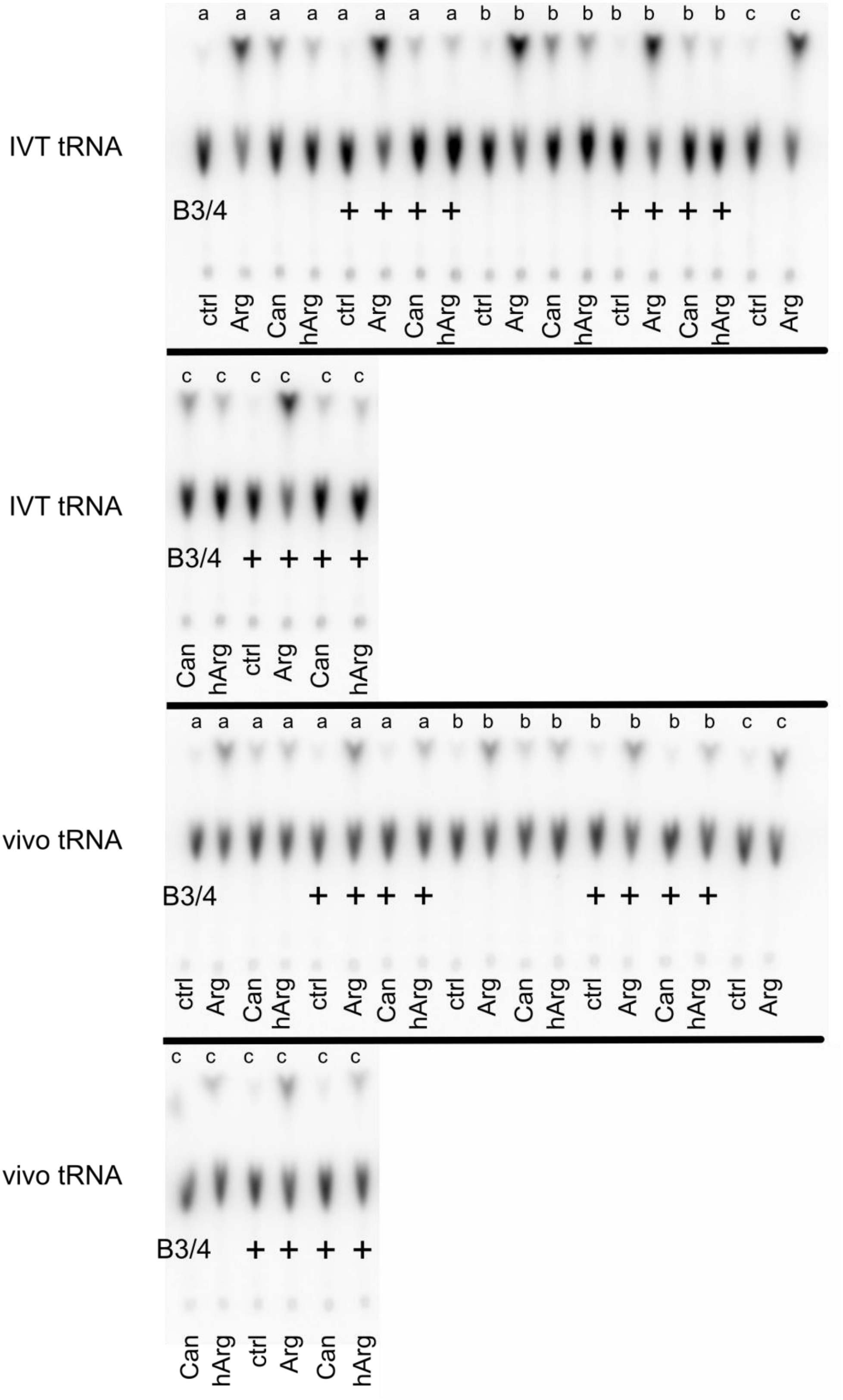

SI Figure 5: Radio screens of TLC separated 3' ^{32}P (aminoacylated) AMP of triplicate experiments (a, b, c). Either *in vitro* or *in vivo* produced tRNA was used in the aminoacylation experiment, which was conducted in the presence (+) or absence ( ) of B3/4 domain like protein from *P. canavaninivorans*. , the substrate used in the reaction is shown beneath the TLC loading spot: ctrl: H_2O, Arg: L-Arginine, Can: canavanine, hArg: homoarginine.

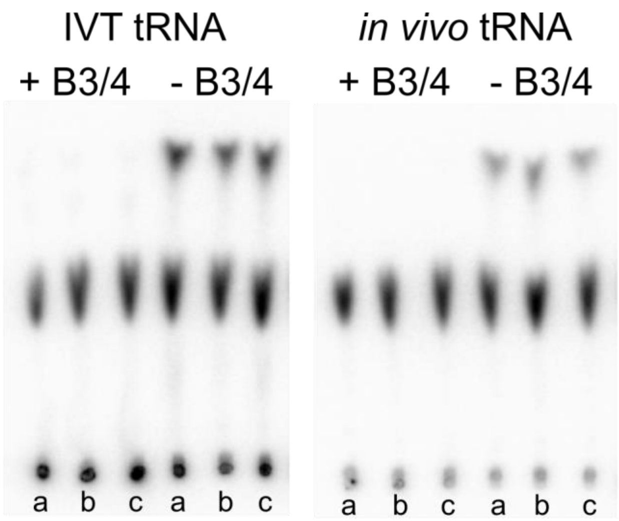

SI Figure 6: Radio screens of TLC separated 3’ ^{32}P labelled (canavanylated) AMP of triplicate experiments (a, b, c). Purified canavanylated $tRNA^{Arg}$ was incubated with or without 50 µM of B3/4 domain like protein from *P. canavaninivorans* for one minute, quenched and then spotted onto a TLC plate.
